# Adaptation and Validation of a Chinese Version of Patient Health Engagement Scale for Patients with Chronic Disease

**DOI:** 10.3389/fpsyg.2017.00104

**Published:** 2017-02-06

**Authors:** Yaying Zhang, Guendalina Graffigna, Andrea Bonanomi, Kai-chow Choi, Serena Barello, Pan Mao, Hui Feng

**Affiliations:** ^1^Xiang Ya Nursing School, Central South UniversityChangsha, China; ^2^Department of Psychology, Università Cattolica del Sacro CuoreMilan, Italy; ^3^Department of Statistical Sciences, Università Cattolica del Sacro CuoreMilan, Italy; ^4^The Nethersole School of Nursing, The Chinese University of Hong KongHong Kong, China

**Keywords:** patient engagement, patient engagement measure, patient health engagement scale, patient activation, psychometric properties

## Abstract

The Patient Health Engagement Scale (PHE-s) was designed to assess the emotional and psychological attitudes of patients' engagement along their healthcare management journey. The aim of this study was to validate a culturally adapted Chinese version of the PHE-s (CPHE-s). Three hundred and seventy-seven participants were recruited from eight community health centers in a sample of patients with chronic disease in Hunan Province, China. The original Italian PHE-s was translated into Mandarin Chinese using a standardized forward–backward translation. The Rasch model was utilized and presented uni-dimensionality and good items fitness of the PHE-s. The internal consistency was 0.89 and the weighted Kappa coefficients of the items (test–retest reliability) ranged from 0.52 to 0.79. Both principal component analysis and confirmatory factor analysis supported a single-factor structure of the PHE-s. In testing the external validity, the PHE-s showed a significant moderate correlation with patient activation but not with medicine adherence behavior, which requires further exploration. The result suggested that the PHE-s is a reliable and valid instrument to assess the level of patient engagement in his or her own health management among chronic patients in China. Further analysis of reliability and validity should be assessed among other patient cohorts in China, and future directions for testing changes after patient engagement interventions should be developed by exploring some clinical relevance.

## Introduction

Chronic diseases are the leading health concerns of the twenty-first century. Due to longer life expectancy and an aging population, control and prevention measures are urgently needed in the healthcare system (Ali et al., 2015). Deaths from chronic diseases rose by just under 8 million between 1990 and 2010, accounting for two-thirds of global deaths, half of all disabilities, and rapidly growing costs (Lozano et al., 2012). The low- and middle-income countries are projected to experience the greatest challenge resulting from chronic disease, which makes up 80% of the causes of death among the world's population (Bloom et al., 2011) and bears more serious burden (Boutayeb, 2006). In China, rapid transitions are also occurring on account of demography and epidemiology (Zhou et al., 2016). According to the latest data from the “2015 Report on Chinese Nutrition and Chronic Disease” released by the National and Family Planning Commission (NHFPC) (http://www.chinadaily.com.cn/m/chinahealth/2015-07/08/content_21224293.htm), the prevalences of hypertension and diabetes among Chinese adults in 2012 were 25.2 and 9.7%, respectively. The incidence rate of cancer was also on the rise, reaching 235 per 100,000 people in 2013. Moreover, 533 out of every 100,000 Chinese residents died from chronic disease in 2012, resulting in 86.6% of all deaths with cardio-cerebrovascular disease, cancer, and chronic respiratory disease as the top causes (Yang et al., 2013).

As a way of combating this growing health crisis, health systems are under a paradigm shift in the planning and delivery of healthcare from patients being viewed as passive recipients of care to being more active and accountable for their own health (Osborn and Squires, 2012). More and more theories and practices have advocated considering patients to be key resources in self-management of chronic diseases. The evidence-based Chronic Care Model illustrated the importance of productive interactions between patients and health practitioners and also highlighted the crucial connection between patient engagement and desirable health outcome (Wagner et al., 2001; Bodenheimer et al., 2002; Wasson and Coleman, 2014). The increasing evidence has also demonstrated that patients' engagement in their own health care process is a core generic components to achieving patient-centered care and successful health management (Ishikawa and Yano, 2011; Miles and Mezzich, 2011). This is particularly true in the case of chronic disease (Simmons et al., 2014). These growing findings in previous serial studies have shown that patients who are more active and engaged in healthcare frequently report improved patient satisfaction and better care experiences, more effective medication management and improved medication safety, higher quality of life, access to health behaviors, and better clinic outcomes, even likely using fewer health care services and contributing to a reduction of healthcare costs (Coulter, 2012; Osborn and Squires, 2012; Hibbard and Greene, 2013; Barello and Graffigna, 2014; Cunningham, 2014; Laurance et al., 2014).

Clearly, patient engagement is perceived to be of importance in the health care system. It is essential to have a good understanding of the level of patient engagement by assessing what works, how it works, and whether engagement efforts are improving outcomes over time (Carman et al., 2013; Wasson and Coleman, 2014). However, to the best of our knowledge, the measurement of patient engagement remains a substantial issue for policy makers and healthcare practitioners at present; few scientifically validated assessment tools exist to help identify patients' level of engaging in their care. Most generic instruments developed in the field of chronic disease—such as the Patient Enablement Instrument designed to capture patients' ability to understand the nature of diseases and copy with their health problems (Howie et al., 1998); the Partners in Health scale, which is a generic assessment scale for patients managing their chronic medical conditions (Battersby et al., 2003); the Patient Activation Measure, which provided an evaluation of individuals' knowledge, skill, and confidence for managing their own health or healthcare (Hibbard et al., 2005); and the Self-Management Ability Scale focused on self-management abilities in relation to well-being (Cramm et al., 2012) can be grouped into one category aimed at assessing the patients' ability to self-manage disease conditions (Eikelenboom et al., 2015). In fact, apart from oriented measurements toward health literacy and behavioral components of patients' self-management, increasing evidence was also accumulating to learn from patients' life stories and call on giving birth to patients' chronic illness trajectory across time (Morales-Asencio et al., 2014), particularly on the emotive component of patient engagement. Those were conceived of as the patients' process elaborating and adjusting to the disease and often appeared to be the first movers of patients' confidence and ability to increase health literacy and reinforce self-management behaviors (Hudson et al., 2014; Graffigna and Barello, 2016). Several existing studies have demonstrated that patients with increased and positive emotion and psychology to attend their own health care are more likely to perform improved patient activation (Hibbard and Mahoney, 2010), enact specific health behavior (Graffigna et al., 2015a), and even have ameliorative health-related biological markers (Ismail et al., 2004). On the contrary, patients are often being debilitated because of emotive disorders, and which may further affect their behavioral choices (Shubin et al., 2015) and may even develop into a vicious cycle (Hibbard and Mahoney, 2010).

However, minimal attention has been given in this aspect to evaluate the emotional and psychological dynamic of patients' engagement experiences along their care management journeys using measures like the Patient Health Engagement Scale (PHE-s), which was recently developed by Graffigna et al. (2015a). Even though the psychometric properties of the PHE-s may not yet be well established, it was still able to be generalized in other countries, because it offered a measure of patient engagement for the first time that more holistically considers the psychological elaboration of the patient based on a rigorous conceptualization model—namely, PHE model. In this model, patient engagement was deemed to be a dynamic and evolutionary process that features four stages: blackout, arousal, adhesion, and eudaimonic project (Graffigna and Barello, 2014; Graffigna et al., 2014). The original Italian version of the PHE-s has only five items and is easy to answer due to its shortness. Each item has seven response options that allow patients to position themselves along a continuum of patient engagement experiences (according to the phases featured by the PHE model). The scale has an ordinal nature and is easy to apply. The instrument can be self-administered by the patient and gives interesting and pragmatic cues to the clinicians in order to best customize their communicational and relational strategies aimed at sustaining patient engagement.

At present, a Chinese version of the PHE-s remains unavailable. Therefore, in the current study, we aimed to translate the original Italian PHE-s into Chinese Mandarin and to evaluate its psychometric properties in a group of patients with chronic disease in China.

## Materials and methods

### Translation and culture adaptation of the PHE-s

In this study, the original version of the PHE-s was translated as recommended by the WHO (World Health Organization) procedures for cross-cultural validation and adaptation of a self-report instrument. The method involved the following steps: (1) forward translation by two bilingual language experts who translated the PHE-s from Italian into English; (2) forward translation by two bilingual language experts who translated the PHE-s from English to Mandarin Chinese; (3) experts' qualitative interviews in order to evaluate semantic and content equivalence, which included eight professionals with broad work experience in chronic care, health research, clinical psychology, and translating; (4) back translation by two additional bilingual experts who translated the Chinese version scale back into English; (5) back translation by two additional bilingual experts who translated the scale from English into Italian while the original author was invited to distinguish the back translation from the English and original versions; and (6) a pilot test among 27 patients with chronic diseases to check the readability and understandability of items and cognitive equivalence of the translation. The final version of the Chinese PHE-s (CPHE-s) was established by consensus and attached online as Appendix I.

### Other instruments

#### Patient activation measure—short form

The American short form of the Patient Activation Measure (PAM13) developed by Hibbard et al. (2005) is an interval-level, unidimensional, Guttman-like scale to provide an assessment of the potential or capacity for patients to be engaged in their health care from three aspects of disease self-management including patient knowledge, skills, and confidence. The response categories of the 13-item scale ranged from strongly disagree to strongly agree and “not applicable.” The raw scores were converted from the continuous Rasch item response theory logit scale to activation scores between 0 and 100 with higher scores indicating greater patient activation. The scale has been widely used and has been shown to be reliable and validated in many different contexts (Maindal et al., 2009; Brenk-Franz et al., 2013; Brucki et al., 2014; Graffigna et al., 2015c; Moljord et al., 2015). In this study, we used the Mainland Chinese version of the PAM13 (PAM13-C) obtained by following the WHO guidelines for cross-cultural adaptation of instruments, and permission for use was obtained from Insignia Health, Inc. The Cronbach's alpha of the PAM13-C was 0.84, indicating good internal consistency among the items. A partial credit Rasch model was used to assess the item fit of the PAM13-C. The item statistics ranged from 0.81 to 1.25 for the infit mean square (MNSQ) and from 0.82 to 1.12 for the outfit MNSQ, suggesting that all the items are productive measurements (Linacre, 2011).

### Morisky medication adherence scale

The Morisky Medication Adherence Scale (MMAS-4) (Morisky et al., 1986) is a 4-item self-reported scale used to assess patients' medication-taking behavior. The MMAS-4 addressed the essential reasons for non-adherence including forgetting, carelessness, and stopping the drug when feeling better or worse. Response categories were yes and no for each item with a dichotomous response. Scores obtained from the MMAS-4 ranged from 0 to 4. Scores of 0, 1 to 2, and 3 to 4 were classified as high, medium, and low adherence, respectively. The Chinese version of the MMAS-4 was available, and the psychometric properties have been established, showing adequate internal consistency with a Cronbach's alpha coefficient of 0.76 for the total scale (Xu et al., 2007; Li et al., 2010).

### Demographic and clinical variables

Participants were asked for information regarding age (continuous), gender, marital status, educational attainment, occupational status, type of diagnosis, and insurance type. Education attainment level was categorized as “primary school and below,” “junior secondary school,” “senior and specialized secondary school,” and “college or higher.”

### Procedure

**Figure 1:**
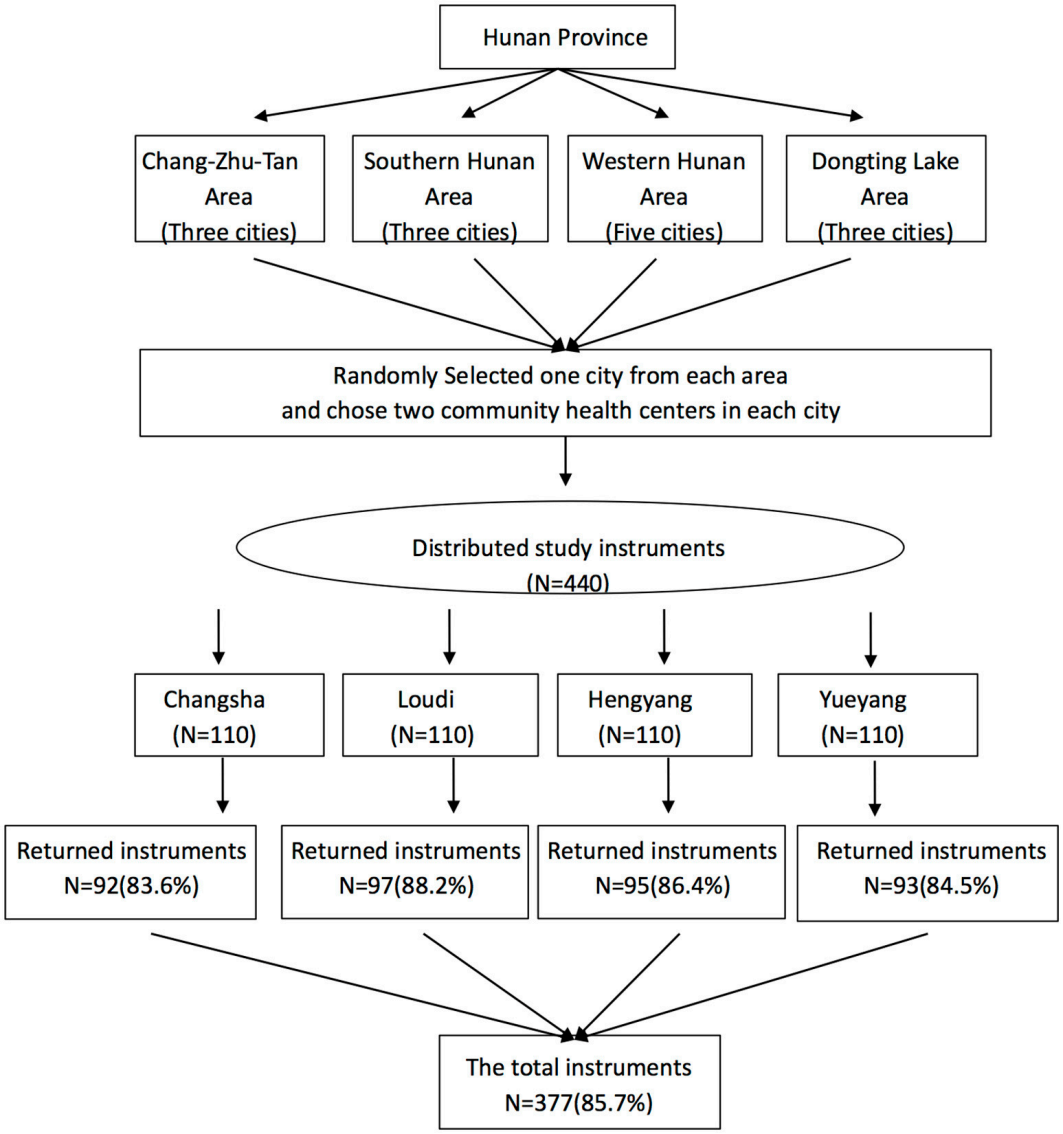
**Flow chart of sample frame and numbers of participants from each city**.

To enhance representativeness, a hierarchical sampling process was employed to recruit participants with chronic illnesses from community health centers in Hunan Province of China. Hunan Province was stratified to four geographic areas, and one city was randomly selected from each area. Data from the Hunan Health Ministry was used to compile a sampling frame of all community health centers in each of the four selected cities. All community health centers that agreed to participate in the study were provided with unique code numbers using SPSS 23.0, and then two community health centers were randomly selected as settings for recruiting participants. One hundred and ten participants were targeted in the two selected community centers in each of the four cities and recruited using a consecutive sampling method if they met the following selection criteria: (1) age > 18 years, (2) diagnosed with one or more chronic disease, and (3) following a chronic treatment for their diseases. Participants with cognitive impairment, uncontrolled psychiatric illness, or serious hearing impairment were excluded from the study. A total of 377 participants were recruited between November, 2015, and February, 2016, and completed the questionnaire; the sample frame and the detailed numbers of participants from each city are presented in Figure 1. Moreover, a sub-sample of 27 participants from one community health center in Changsha completed a test–retest evaluation of the CPHE-s through a telephone investigation after a 2-week interval.

## Ethics statement

Permissions were obtained from the authors and copyright owners of the original scale development research. The study was approved by the Ethical Committee of Xiang Ya Nursing School, Central South University in China. All participants received a short study description and were asked to give oral permission to take part. Patients were also informed about anonymity and their right to withdraw from the study at any time without consequences. The results were analyzed at the group level and for scientific purposes exclusively. There are no vulnerable populations in this study.

## Data analysis

The data were entered by EpiData 3.02 and analyzed using IBM SPSS 23.0, Amos 23.0, and R 3.2.4. Appropriate descriptive statistics for ordinal data were used to summarize and present the data. The content validity was assessed through the application of the content validity index (CVI), and the item-level CVI (I-CVI) and Scale-level Index Average (S-CVI/Ave) were reported to compare the relevance of the translated Chinese version with the English version in this study (Polit and Beck, 2012). The partial credit Rasch model specifying additivity and uni-dimensionality was employed to assess the item fit of the CPHE-s. The infit MNSQ statistic was used to assess the item fit. An infit value of an item falling between 0.5 and 1.5 indicates that the item is productive for the underlying measurement (Linacre, 2011). The internal consistency of the items of the CPHE-s was assessed using Ordinal Alpha via Empirical Copula Index (Bonanomi et al., 2012, 2015) and inter-item polychoric correlation given the ordinal nature of the items. A reliability index ≥ 0.7, 0.8, or 0.9 can be interpreted as acceptable, good, or excellent, respectively (Gliem and Gliem, 2003). Test–retest reliability of the instrument was assessed using weighted kappa. Weighted kappa values can be interpreted as follows: ≤0.20 as poor, 0.21–0.40 as fair, 0.41–0.60 as moderate, 0.61–0.80 as substantial, and >0.8 as almost perfect (Altman, 1991). Principal component analysis (CATPCA) was performed to identify the factor structure of the polytomous items of the CPHE-s. Confirmatory factor analysis (CFA) was performed to study the replicability of the factor structure obtained by CATPCA. The estimation method was asymptotically distribution free, particularly suitable for ordinal data and not-Gaussian distributions. Measurement invariance by gender was also investigated. Finally, to assess the external validity of the CPHE-s, we evaluated the correlations between patient engagement (CPHE-s) and the PAM13 as well as medication adherence (MMAS-4) using SPSS 23.0 with Pearson's correlation coefficient. We expected high positive correlations between the CPHE-s and PAM13 as well as the MMAS-4. Significance level was set to α = 0.05.

## Results

### Translation and adaptation

During the translation process, some modifications were necessary to address cultural differences between Western and Eastern countries. For example, the word “blackout” refers to a sudden status of power outages or flameout that is too abstract to understand for Chinese, especially elders with chronic disease. Therefore, we translated “I feel like I have blacked out” into “I feel my mind/brain goes blank.” Further, there is no obvious difference between “aware” and “conscious,” which both illustrate a status of attitude or cognition. “I have gained some understanding gradually” and “I know my disease/status” were used to distinguish two different intensities. In the current study, an expert panel including three clinicians experienced in chronic care or psychology from university teaching hospitals and two faculty members from university was asked to rate each item of the CPHE-s. The I-CVI was between 0.80 and 1.00, and the S-CVI/Ave was 0.92 in the final version of the CPHE-s. These results indicate acceptable content validity for the CPHE-s. More detailed information of the forward and back translations is presented in Appendix II.

### Sample

Among 440 chronic patients who participated in the current study, 377 completed the survey for the psychometric analysis for an overall response rate of 85.7%. The most common reasons for refusal were lack of time and poor physical condition on the day of the survey. Demographic and clinic characteristics of the participants are summarized in Table 1. It was noted that, for these clinic diagnoses, the groups were not independent since most of the participants had more than one chronic disease. Additionally, 27 out of 30 participants from a randomly selected sub-sample completed the test–retest evaluation of the CPHE-s after a 2-week interval. Of those, 59.3% were women, and 40.7% were men with a mean age of 53.8 years (*SD* = 11.0) and a range of 34–78 years.

**Table 1:** **Demographic and clinical characteristics of the sample (*n* = 377)**.

**Characteristics**	**n (%)**
**AGE (YEARS)**	
< 60	107 (28.4)
60–74	182 (48.3)
≥ 75	88 (23.3)
**GENDER**	
Male	172 (45.6)
Female	205 (54.4)
**MARITAL STATUS**	
Never married	1 (0.3)
Married	316 (83.8)
Divorced	6 (1.6)
Windowed	54 (14.3)
**EDUCATION**	
Primary school and below	122 (32.4)
Junior secondary school	124 (32.9)
Senior and specialized secondary school	80 (21.2)
College or higher	51 (10.75)
**EMPLOYMENT**	
Student	1 (0.3)
Unemployed	131 (34.7)
Retired	209 (55.4)
Employed	36 (9.5)
**INSURANCE TYPE**	
UEBMI	208 (55.2)
URBMI	122 (32.4)
NRCMS	45 (11.9)
Uninsured	2 (0.5)
**CHRONIC DISEASES(%)**	
Hypertension	271 (71.9)
Diabetes	110 (29.2)
Cerebrovascular disease	50 (13.3)
Cardiovascular disease	102 (27.1)
COPD	41 (10.9)
Cancer	15 (4.0)
Rheumatoid arthritis	9 (2.4)
Osteoarthritis	21 (5.6)
Osteoporosis	1 0.3)
Thyroxine disorder	5 (1.3)
Uremia	4 (1.1)
Asthma	1 (0.3)
Hepatitis	6 (1.6)
Hypercholesterolemia	9 (2.4)
Depression	1 (0.3)

*UEBMI, Urban employee basic medical insurance; URBMI, Urban residents' basic medical insurance; NRCMS, New rural cooperative medical system*.

### Responses to the chinese patient health engagement scale (CPHE-s)

Since the Pearson correlation between the ordinal factorial score and median is very high (equal to 0.86), the median of the CPHE-s is considered to be a robust and efficient estimator of the real latent score of the construct—namely, the level of patient engagement. Table 2 provides the item-level descriptive statistics for all items. Given the ordinal nature of the items, the median and the Shannon Entropy Index were calculated as tendency central and dispersion indices, respectively. Furthermore, there was no severe floor or ceiling effect for the summary score of the CPHE-s since only 17 (4.5) and 44 (11.7%) of the participants achieved the lowest and highest possible scores, respectively.

**Table 2:** **Item-level descriptive statistics for ranks on the CPHE-s**.

**CPHE-s item**	**Rank range**	**Minimum**	**Maximum**	**Median**	**Shannon entropy**
Item 1	1–4	1	4	3	0.77
Item 2	1–4	1	4	3	0.77
Item 3	1–4	1	4	3	0.81
Item 4	1–4	1	4	3	0.84
Item 5	1–4	1	4	3	0.83

### Item analysis

A Partial Credit Rasch Model was implemented to further investigate whether the CPHE-s was uni-dimensional and whether all items fit the model well. Infit MNSQ statistic was computed to check whether the items fit the expected model. MNSQ determines how well each item contributes to defining a single underlying construct (uni-dimensionality). Infit is more sensitive to misfit responses to items closest to the person's ability level.

The logit measures and infit MNSQ statistics of the CPHE-s items are given in Table 3. The logit measures of the items ranged from 0.47 to 0.50, indicating that the item difficulties are not greatly varied. None of the items had infit MNSQ statistics < 0.5 or > 1.5, suggesting that all the items are productive for the underlying measurement (Linacre, 2011). The person separation index (PSI) was calculated to evaluate the reliability in the Rasch Model (PSI = 0.884).

**Table 3:** **Logit measures and mean square Infit statistics for the partial credit Rasch model of the CPHE-s**.

**CPHE-s**	**Logit measure (error)**	**Infit MNSQ**
Item 1	0.11 (0.16)	1.43
Item 2	−0.47 (0.16)	1.22
Item 3	0.50 (0.16)	0.79
Item 4	−0.13 (0.16)	0.74
Item 5	0.00 (0.16)	0.77

## Reliability

### Internal consistency and test–retest reliability

The CPHE-s had very good internal consistency since the value of the Ordinal Alpha via Empirical Copula was equal to 0.89. In Table 4, the Ordinal Alpha was evaluated after deleting individual items. Each item contributed significantly to the PHE-s score. The internal consistency of the CPHE-s was satisfactory. The Ordinal Alpha if item is deleted and weighted kappa coefficients (test–retest reliability) are shown in Table 4. The weighted Kappa coefficients of the items ranged from 0.52 to 0.79, suggesting that the strength of agreement of the test–retest values of the CPHE-s items could be interpreted as moderate to good (Altman, 1991).

**Table 4:** **Reliability indices for the CPHE-s**.

**CPHE-s item**	**Ordinal alpha if item deleted**	**Test–retest reliability weighted kappa**
Item 1	0.88	0.74
Item 2	0.87	0.65
Item 3	0.86	0.52
Item 4	0.85	0.53
Item 5	0.85	0.79

### Internal structure

#### Exploratory analysis

The sample has been divided randomly into two sub-groups with an exclusive and exhaustive procedure: Group 1 (*n* = 227, 55.9% women, aged 36–90 years old; *M* = 66.1 years, *SD* = 9.8) for exploratory analysis and Group 2 (*n* = 150, 52% women, aged 26–87 years old; *M* = 65.4 years, *SD* = 10.6) for confirmatory analysis.

An exploratory categorical CATPCA was conducted on the final CPHE-s in Group 1 because of the ordinal nature of the items. An initial analysis was performed without any restriction on the number of metric factors to be estimated. The initial analysis yielded one factor with eigenvalue 4.1, explaining 87.1% of the total variability. Table 5 shows the factor loadings for the one solution of the CATPCA. All factor loadings had a very high value (>0.8).

**Table 5:** **Factor loadings from CATPA – One factor solution**.

**CPHE-s item**	**One factor solution**
Item 1	0.74
Item 2	0.71
Item 3	0.84
Item 4	0.89
Item 5	0.88

All the conducted analyses (Rasch Model, Item fit analysis, PSI, Ordinal Alpha, eigenvalue, and explained variability of the first component of CATPCA) confirmed the uni-dimensionality of the scale. Table 6 gives the inter-item polychoric correlation matrix of the CPHE-s. The average inter-item polychoric correlation is a subtype of internal consistency reliability. It is obtained by taking all the items on a test that probes the same construct, determining the polychoric correlation coefficient for each pair of items, and finally taking the average of all of these polychoric correlation coefficients. All inter-item polychoric correlations were higher than 0.7, indicating good inter-correlation between the items. The average inter-item polychoric correlation is equal to 0.84, indicating a high correlation between items.

**Table 6:** **Polychoric correlation matrix for the items of CPHE-s**.

**CPHE-s**	**Item 1**	**Item 2**	**Item 3**	**Item 4**	**Item 5**
Item 1	–	0.94	0.79	0.75	0.72
Item 2		–	0.80	0.81	0.79
Item 3			–	0.93	0.90
Item 4				–	0.98
Item 5					–

### Confirmatory analysis

**Figure 2:**
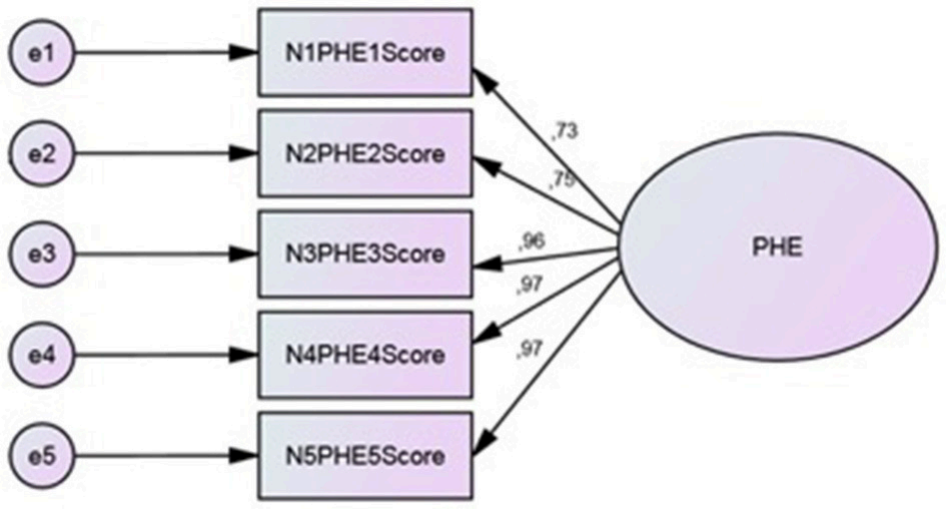
**CFA on CPHE-s: Standardized estimates**.

CFA (Figure 2) was performed on Group 2 to study the replicability of the factor structure obtained by CATPCA. The estimation method was asymptotically distribution free, particularly suitable for ordinal data and not-Gaussian distributions. To evaluate the closeness of the hypothetical model to the empirical data, multiple goodness-of-fit indexes were used, including the ratio of the chi-square to degrees of freedom (χ^2^/*df*), the Comparative Fit Index (CFI), the Standardized Root Mean Square Residual (SRMR), the Goodness of Fit Index (GFI), and the Root Mean Square Error of Approximation (RMSEA). To test the model, each variable was allowed to load on only one factor, and one variable loading in the latent factor was fixed at 1.0. For the remaining factor loadings, residual variances were freely estimated.

CFA showed reasonable goodness of fit indices. The fit indices met the criteria of fit for the hypothesized one-factor structure. Chi square (*c*^2^ = 6.65, *df* = 4, *p* = 0.156) value and goodness of fit indices (CFI = 0.983, SRMR = 0.014, GFI = 0.979, RMSEA = 0.067) suggested that the model is coherent with the data. The analysis of modification indices did not suggest any relation between the error covariance of the items, avoiding overlapping problems.

To verify the validity and generalizability of the factor structure, a multi-group confirmatory analysis tested measurement invariance in the two subsamples divided by gender. Table 7 shows the verified invariance hypothesis. The Δχ^2^ between the unconstrained and constrained models did not yield significant results. The factor structure was invariant by gender.

**Table 7:** **Multigroup CFA by gender**.

**Model**	***χ^2^***	***Df***	**RMSEA**	**CFI**	**Δ*χ^2^* (*df*)**	***P***
Unconstrained	6.6	4	0.059	0.971	–	–
Invariant factor loading	21.6	16	0.048	0.994	14.9 (12)	0.25

### External validity

The external validity of the CPHE-s was assessed by correlating the median score of the scale with the PAM-13 and MMAS-4 scores using Pearson's correlation coefficient. A moderate correlation was found between the CPHE-s and PAM-13 (*r* = 0.43, *p* < 0.001). However, there was no significant correlation between the CHPE-s and MMAS-4 (*r* = −0.04, *p* = 0.464).

## Discussion

This cross-sectional study provided the first report on the translation and validation of the PHE-s into the Chinese Mandarin language. The Rasch analysis presented a series of good infit values (ranging from 0.74 to 1.43) for each item of the CPHE-s. The data demonstrated good internal consistency of the PHE-s for patients with chronic disease through satisfactory Ordinal Alpha (α = 0.89), which is higher than the original Italian Cronbach coefficient (α = 0.85) (Graffigna et al., 2015a), and good test—retest analysis (the weighted Kappa coefficients of the items ranging from 0.52 to 0.79) (intra-class correlation coefficient = 0.68), which is lower than the value of the Italian measure (Graffigna et al., 2015a) but is still accepted. The exploratory categorical CATPCA and CFA suggest that the PHE-s belongs to a single-factor and uni-dimensionality scale.

To assess the external validity of the CPHE-s, the CPHE-s factor scores were first evaluated in relation to the PAM13 with strong psychometric properties as a golden standard, and the Pearson's correlation coefficient was 0.43, which is consistent with the Italian finding (Graffigna et al., 2015a). With some degree of conceptual overlapping, the terms “patient engagement” and “patient activation” are often used interchangeably. Patient activation contributed to describe the degree of patient engagement as an active agent in managing their own health, and higher levels of activation have been associated with greater patient engagement in health care (Carman et al., 2013; Hibbard and Greene, 2013; Graffigna et al., 2015a,b; Menichetti et al., 2016).

In contrast to the Italian data (Graffigna et al., 2015a), the Chinese results failed to support significant and negative associations between PHE and MMAS-4 scores. One plausible explanation for this finding stems from previous studies (Young et al., 2014; Awwad et al., 2015) that suggest that medicine adherence reports may tend to be skewed in favor of reporting higher adherence. Some responders might not disclose non-adherence as it might be deemed undesirable behavior, especially in the face-to-face investigation. Another explanation for this finding is that a four-item scale is not sufficient to represent the entire domain of the medication adherence construct (Morisky and DiMatteo, 2011). The eight items of the MMAS, which were shown to have higher reliability than the original four-item scale (Morisky et al., 2008), should be taken into consideration to further explore the correlation between patient health engagement level and medication adherence in future research.

Concerning potential shortcoming and limitations, first, this study is liable to recall bias like other studies based on self-reported measures, because it is hard to differentiate between patients who have actually addressed a high level of patient engagement and those reporting a high level of patient engagement for social desirability. Second, the relative heterogeneity of samples may be regarded as a weakness. Participants enrolled in community health centers represent a wide range of patients with chronic diseases, including patients with acute care, patients undergoing routine examinations, and patients with multi-morbidity. This might be negatively affected and lead to some errors in concurrent analysis like the relationship between patient health engagement and medicine-taking behaviors. Hence, further research is warranted to confirm the validation of the CPHE-s in a stratified manner representative of the Chinese chronic population.

In conclusion, our research adds to the accumulating evidence that the PHE-s has good validity and reliability in the context of Eastern culture. Healthcare practitioners can use it in primary care settings to better understand the patient engagement levels among patients taking part in their own health management. Differences in relevance between patient health engagement and medication-taking behavior require further investigation considering a revised MMAS-8 or other questionnaires and some objective indexes such as pharmacy refill records and pill counts (Santra, 2015).

## Author contributions

YZ was responsible for the literature search, analysis, data collection and coordination, interpretation of the data, drafting, writing, and revising the work. All authors contributed to the design (GG, SB, HF), data collection and coordination (PM, HF), analysis and interpretation of data (AB, KC), and/or writing and revising the work critically for important intellectual content (GG, AB, SB, HF). All authors read and approved the final manuscript and agree to be accountable for all aspects of the work in ensuring that questions related to the accuracy or integrity of any part of the work are appropriately investigated and resolved.

### Conflict of interest statement

The authors declare that the research was conducted in the absence of any commercial or financial relationships that could be construed as a potential conflict of interest.

## Abbreviations

PHE: patient health engagement
PHE-s: patient health engagement scale
CPHE-s: The Chinese version of patient health engagement scale.
