# Supplementary material for: Adaptation and Validation of a Chinese Version of Patient Health Engagement Scale for Patients with Chronic Disease
Source: Front Psychol. 2017 Feb 6;8:104. doi: 10.3389/fpsyg.2017.00104 (PMC5292425; doi:10.3389/fpsyg.2017.00104)
Supplement: Supplementary file 1 [file Table1.DOCX]

**Appendix I The Chinese version of Patient Health Engagement Scale (CPHE-s)**

**患者健康参与量表**

以下是有关人们谈论自己健康状况时的个人体验，请依据您个人的实际状况，在每个选项中**圈出最能准确表达您当前感受状态的分值**。

| **当我想起自己的疾病（或健康状况）时：** | | | | |
| --- | --- | --- | --- | --- |
| **1** | 我感觉大脑一片空白 | 我很警觉 | 我渐渐有所了解 | 我感到积极乐观 |
|  | **1 2 3 4 5 6 7** | | | |
| **2** | 我感到茫然不知所措 | 我很烦恼 | 我了解自己的健康状况（疾病） | 我感到很平静 |
|  | **1 2 3 4 5 6 7** | | | |
| **3** | 一旦想起自己的疾病，我感到很崩溃 | 每当出现新的症状时，我就会变得焦虑 | 我已经习惯了自己的病情 | 尽管我生病了，我觉得生活并没有发生改变 |
|  | **1 2 3 4 5 6 7** | | | |
| **4** | 我对自己的疾病感到很沮丧 | 当我尝试管理自己的疾病时，我感到焦虑 | 我已经适应了自己的病情 | 尽管我生病了，我觉得生活仍将继续 |
|  | **1 2 3 4 5 6 7** | | | |
| **5** | 我感觉自己完全被疾病所折磨 | 当新的症状出现时，我很沮丧 | 我已经接受了自己的疾病 | 尽管我生病了，我也能活的有意义 |
|  | **1 2 3 4 5 6 7** | | | |
