# Supplementary material for: Adaptation and Validation of a Chinese Version of Patient Health Engagement Scale for Patients with Chronic Disease
Source: Front Psychol. 2017 Feb 6;8:104. doi: 10.3389/fpsyg.2017.00104 (PMC5292425; doi:10.3389/fpsyg.2017.00104)
Supplement: Supplementary file 2 [file Table2.DOC]

Appendix Ⅱ Forward and back translation of the Chinese version of Patient Health Engagement scale (CPHE-s)

| No | Original version | Chinese version refined | Back translated version |
| --- | --- | --- | --- |
| 0 | Thinking about my health status… | 当我想起自己的疾病（或健康状况）时： | When I think of my disease (health problem): |
| 1 | I feel in blackout. | 我感觉大脑一片空白。 | I feel my mind/brain goes blank. |
|  | I am in alarm. | 我很警觉。 | I am very alert/ vigilant. |
|  | I am aware. | 我渐渐有所了解。 | I have gained some understanding gradually. |
|  | I feel positive. | 我感到积极乐观。 | I feel optimistic and positive. |
| 2 | I feel dazed. | 我感到茫然不知所措。 | I feel lost. |
|  | I am in trouble. | 我很烦恼。 | I feel I am in trouble. |
|  | I am conscious. | 我了解自己的健康状态（疾病）。 | I know my disease/ status. |
|  | I feel serene. | 我感到很平静。 | I feel calm/ serenity. |
| 3 | When I think about my illness I feel overwhelmed by emotions. | 一旦想起自己的疾病，我感到很崩溃。 | Once I think of my disease,I feel I’m near the point of breakdown/ collapse. |
|  | I feel anxious every time a new symptom arises. | 每当出现新的症状时，我就会变得焦虑。 | When new symptoms occur, I become anxious. |
|  | I got used to my illness condition. | 我已经习惯了自己的病情。 | I adapt myself to my situation. |
|  | Despite my illness  I perceive coherence and continuity in my life. | 尽管我生病了，我觉得生活并没有发生改变。 | Despite my illness, my life does not change much. |
| 4 | I feel very discouraged due to my illness. | 我对自己的疾病感到很沮丧。 | I feel upset with my disease. |
|  | I feel anxious when I try to manage my illness. | 当我尝试管理自己的疾病时，我感到焦虑。 | When I try to manage my disease, I feel anxiety. |
|  | I feel I adjusted to my illness. | 我已经习惯了自己的病情。 | I get used to my own condition. |
|  | I am generally optimist about my future and my health condition. | 尽管我生病了，我觉得生活仍将继续。 | Though I am ill, I feel life has to move on in spite of my illness. |
| 5 | I feel totally oppressed by my illness. | 我感觉自己完全被疾病所折磨。 | I feel I am completely tortured by my illness. |
|  | I am upset when a new symptom arises. | 当新的症状出现时，我很沮丧。 | I am very depressed when new symptoms occur. |
|  | I feel I have accepted my illness. | 我觉得我已经接受了自己的疾病。 | I think I accepted my disease. |
|  | I can give sense to my life despite my illness condition. | 尽管我生病了，我也能活的有意义。 | Even if I am ill, I may live a meaningful life as well. |
